# Unraveling the molecular basis of snake venom nerve growth factor: human TrkA recognition through molecular dynamics simulation and comparison with human nerve growth factor

**DOI:** 10.3389/fbinf.2025.1674791

**Published:** 2025-10-24

**Authors:** Shrudhi Devi, Gurunathan Jayaraman

**Affiliations:** School of Biosciences and Technology, Vellore Institute of Technology, Vellore, Tamil Nadu, India

**Keywords:** snake venom, nerve growth factor, tyrosine receptor kinase A, neurodegenerative disease, simulation

## Abstract

**Introduction:**

Neurodegenerative diseases pose significant challenges owing to the limited number of effective therapies. Nerve growth factor (NGF) plays a crucial role in neuronal survival and differentiation through tropomyosin receptor kinase A (TrkA). Although snake venom NGF (sNGF) has been studied for its ability to activate TrkA, the binding modes and associated dynamics remain unclear compared to those of human NGF (hNGF). Herein, we explored the possibilities of NGFs from *Daboia russelii* and *Naja naja* as potential therapeutic alternatives to hNGF by comparing the structural similarities and conserved binding residues.

**Methods:**

The active sites were identified through a literature review, molecular docking was performed using HADDOCK, and molecular dynamics simulation was performed to analyse the stabilities of the complexes; then, PRODIGY and molecular mechanics Poisson–Boltzmann surface area were used to determine the binding affinities.

**Results:**

The different sNGFs exhibited stronger binding affinities and stabilities than hNGF, while principal component analysis and the free energy landscape indicated constrained conformational flexibilities suggestive of an adaptive mechanism in sNGF for effective receptor engagement. A network coevolutionary analysis was performed, which showed the pattern in which the amino acids were coevolved and conserved throughout the simulations.

**Discussion:**

These findings indicate that NGFs from *D. russelii* and *N. naja* are promising therapeutic candidates for treating neurodegenerative disorders and warrant further *in vivo* validation.

## Graphical Abstract

**Figure:**
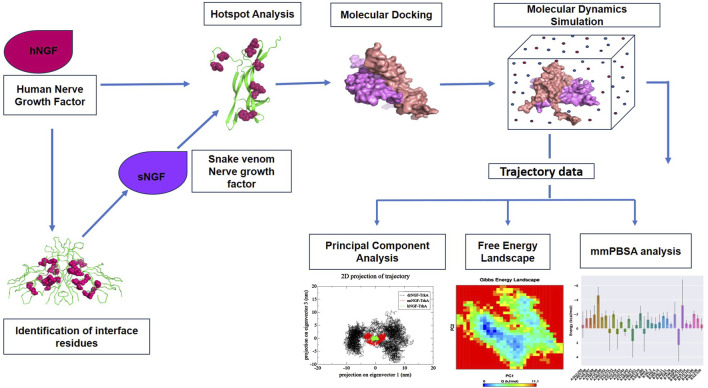

## 1 Introduction

Neurodegeneration is a condition associated with ageing and is primarily caused by the gradual decline of cholinergic neurons as a result of inflammatory processes or the inability of neurons to effectively mediate signals (Aisen et al., 2001; Schmidt et al., 2021). Alzheimer’s disease (AD) is one of the popular neurodegenerative conditions and is characterised by the development of significant behavioural, motor, and cognitive deficits (Godoy et al., 2014). Nerve growth factor (NGF) often acts as a double-edged sword and can either induce neurogenesis by binding with tropomyosin receptor kinase A (TrkA) or induce apoptosis by binding to p75 (p^75NTR^) (Wang et al., 2014; Bruno et al., 2009; Allard et al., 2012; 2018; Iulita et al., 2014; 2016; Wang et al., 2014; Pentz et al., 2021).

The therapeutic potential of human NGF (hNGF) has already been tested for several neurodegenerative conditions, among which the prominent ones are AD (Jönhagen et al., 1998; Aloe et al., 2012), Parkinson’s disease (PD) (Olson et al., 1991; 1992), and traumatic brain injury (Holtzman et al., 1996). However, the potential limitations of using hNGF include insufficient oral bioavailability, uncertain pharmacokinetics, suboptimal pharmacological characteristics, limited capacity to penetrate the blood–brain barrier (BBB), shortened half-life, activation of numerous receptors, and multiple effects that still need to be evaluated. The crosstalk between TrkA and p75, their allosteric effects, and their downstream outcomes are not fully understood. The side effects of NGF administration leading to hyperalgesia have limited its clinical usage (Bernardes et al., 2018; Zhou et al., 2023). Snake venom NGF (sNGF) has been studied for chondrogenesis, neurite outgrowth, neuroprotection, and tumour growth inhibition (Katzir et al., 2003; Osipov et al., 2014; Martins et al., 2015; Lu et al., 2017). As NGF is bioavailable, it activates TrkA and also triggers some anti-apoptotic pathways (Bcl-2, MAPK) to enhance neuronal plasticity (McCleary and Kini, 2013; Bernardes et al., 2018; Gudasheva et al., 2019). Although sNGFs have been reported to possess neurogenic activities, there are limitations in understanding the dynamics of the complex formation between NGF and TrkA as well as mapping of the conformational changes that lead to signalling. Peptides from *Bothrops atrox* (Glu-Val-Trp) and *Daboia russelii* have also been reported to exhibit neuritogenesis as well as protect against oxidative stress and neurotoxicity in PC12 and human neuronal cells; they can also protect from MPP^+^ toxicity by activating TrkA and the anti-apoptotic pathways (Bernardes et al., 2018), while enhancing neuronal plasticity by regulating GAP-43 and synapsin. Similarly, NGF isoforms as well as peptides from *D. russelii* and *B. atrox* can induce neurite outgrowth via TrkA activation and protect against paraquat-induced neurotoxicity by reducing reactive oxygen species (ROS) and apoptosis (Bernardes et al., 2018; Madhubala et al., 2023).

Understanding the roles of sNGFs in neurite outgrowth and neurite protection may help advance the management/treatment of neurodegenerative diseases as novel neurotrophic therapies. The molecular mechanisms behind the binding of the NGFs to TrkA are pivotal for investigating the associated neuroprotective potentials. Considering the similarities in structures and functions between hNGF and sNGF, we provide insights into the molecular evidence of their interactions. Although there are studies that have utilised NGF from *Vipera russelli russelli* for modelling and docking with TrkA, the dynamics and stability of the complex are not yet investigated (Islam et al., 2021). In the present study, we provide molecular evidence of the interactions and stabilities between sNGFs and TrkA as well as information about maintenance of the interacting residues throughout the simulation period. These are particularly important since sNGFs are structurally similar but evolutionarily and functionally distinct; snake venom proteins are also known for their enhanced stabilities owing to the extensive disulphide bonds, protease resistance, and adaptation to the extracellular environment (Sunagar et al., 2013).

The present study aims to provide molecular evidence of the interactions between sNGFs and TrkA through molecular docking and molecular dynamics simulations to assess their stabilities and functional relevance as the binding affinities do not always correlate with the stabilities. By elucidating the binding efficiency, specificity, and stability, we aim to provide a foundation for future investigations into the therapeutic applications of sNGFs in neurodegenerative disorders. To the best of our knowledge, this is a pilot effort on comparative analysis using molecular dynamics and energy decomposition to examine how sNGFs and hNGF bind to TrkA; our findings are expected to provide new insights into the mechanisms behind these high-affinity interactions.

## 2 Materials and methods

### 2.1 Sequence analysis

NGF sequences for both sNGFs and hNGF were retrieved from the UniProt database (Consortium et al., 2025); although there were 112 sNGF sequences in the database, only those sequences that were reviewed by UniProt were considered in this work. Information about the conserved residues of the NGF superfamily was obtained by multiple sequence alignment using the Clustal Omega tool (Sievers et al., 2011), and pairwise alignment was performed using EMBOSS Needle to check the similarity and identity indexes between the selected sequences and hNGF to reveal the structural, functional, and coevolutionary relationships (Rice et al., 2000). After conducting the pairwise alignments of all the sNGF species, mature sequences of only *D. russelii* NGF (drNGF) and *Naja naja* NGF (nnNGF) were used for further studies. As the three-dimensional structures of sNGFs have not been determined experimentally, predicted structures from AlphaFold were used for the structural analyses. The structure of hNGF with TrkA was obtained from RCSB-PDB (1WWW) (Wiesmann et al., 1999).

### 2.2 Physicochemical properties of the proteins

Various tools have been employed to study the characteristics of the proteins, such as stability, half-life, hydrophobicity, and pI, using the tool ProtParam (Gasteiger et al., 2005). InterPro was used to predict the conserved and signature sequences of the NGF (Blum et al., 2025), while Radar was used to predict the repeats (Heger and Holm, 2000) and Pfam was used to identify the functional characteristics of the proteins (Paysan-Lafosse et al., 2025).

### 2.3 Coevolutionary analysis

The coevolutionary analysis of drNGF, nnNGF, and hNGF was conducted using EVcouplings server (http://evfold.org); this tool identifies the amino acid pairs within a protein that have maintained their interactions despite the evolutionary mutations. Even when mutations occur, these interactions remain stable, indicating their importance in the structure and functions of the protein. The analysis is based on preservation of the correct folded conformation of the protein within homologous proteins, such that the same can be explicated even in an invariant folded state structure. We used the JackHMMER algorithm to search the UniProt database, pseudo-likelihood maximisation direct coupling analysis for the evolutionary coupling inference, average product correction for noise removal, and Rosetta or Modeller to generate the structures from the predicted contacts. The closest distance cutoff for true-positive residue contacts between the atoms was 5 Å (Wang et al., 2020; Zhang et al., 2022). To identify the crucial coevolutionary relationships, a residue–residue network was built using the coevolutionary coupling score. The potential structural and functional relevances of the key coevolved clusters were then analysed and visualised using Cytoscape (Shannon et al., 2003).

### 2.4 HotSpot analysis

HotSpot analysis was performed for the hNGF-TrkA complex using the PPCheck, SpotOn, and DrugScore PPI tools; this is an *in silico* alanine-scanning approach in protein–protein interfaces where each of the amino acids at the protein–protein interfaces are substituted with the amino acid alanine to check for binding free energy differences between the wild-type protein structure and alanine mutants based on the knowledge-based quantitative structure–activity relationship approach (Krüger and Gohlke, 2010; Sukhwal and Sowdhamini, 2013; Melo et al., 2016).

### 2.5 Molecular docking

Protein–protein interaction studies were carried out between the NGFs (drNGF/nnNGF/hNGF) and TrkA (extracellular domain: domain-5) using HADDOCK, which is an information-driven flexible docking approach. The models were then sorted on the basis of the energy values and HADDOCK scores; models with the least energy after energy minimisation and clustering based on the root mean-squared distance (RMSD) values as well as their correlations to literature based on the interacting residues were selected for further analyses. The input parameters used for HADDOCK were as follows: accessible residues excluding the active residues were selected such that they had a minimum of 15% relative solvent accessibility and a maximum radial distance of 6.5 Å from the active site residues. The surface neighbouring residues were selected based on the criterion that they must have a relative solvent accessibility of at least 40%. Thus, the active site residues that were considered for the study were H4, E11, W21, R59, H84, and R103. After removing the non-polar hydrogen atoms, the proteins were rotated 180° with water as the solvent for alternate binding orientations that may have been missed otherwise. The clustering method was based on the fraction of common contacts with 0.6 as the RMSD cutoff and 4 as the minimum cluster size. OPLSX was used for the non-bonded parameter calculations of the accurate interaction energies. The electrostatic energy and distance-dependent (rdie) dielectric parameter were set for the energy and interaction parameters. To analyse the hydrogen bonds (proton-acceptor) and hydrophobic interactions (carbon–carbon), the interatomic distances were set to 2.5 and 3.9, respectively (Dominguez et al., 2003; Van Zundert et al., 2016).

### 2.6 Determination of the dissociation constant and affinity

The protein binding energies were predicted using the protein binding energy prediction (PRODIGY) web server (http://haddock.science.uu.nl/services/PRODIGY), which enables users to understand the binding affinities in biological complexes as well as identify protein interfaces between these complexes based on the crystallographic structures; the binding affinities themselves are based on the structural characteristics of the proteins. The value of the dissociation complex (K_d_) was calculated at 25 °C (Honorato et al., 2021).

### 2.7 Molecular dynamics simulation

Molecular dynamics simulations offer a practical method of analysing the stability, folding, and flexibility characteristics of protein–protein complexes in biomolecular systems. The docked (drNGF-TrkA, nnNGF-TrkA, and hNGF-TrkA) complexes were subjected to molecular dynamics simulations using GROMACS (version 2020.2) (Kohnke et al., 2020), and the topologies were delineated using the CHARMM27 all-atom force field. The proteins were contained in cubic boxes of dimensions 7 × 7 × 7 nm each and placed at a distance of 2.0 nm from the box edge. The box was then solved with the SPC 216 model, and charge neutralisation was performed by adding 0–8 Na^+^ and 0–4 Cl^−^ ions as counter ions to the NGF-TrkA system.

The system was next subjected to minimisation using the steepest descent algorithm until a maximal force of <1,000 kJ/mol/nm was achieved. The NVT and NPT equilibrations of the systems were then conducted at 300 K for 100 ps. After equilibration, the molecular dynamics simulations were performed with step sizes of 2 fs for 100 ns. The large-scale electrostatic interactions were calculated using the particle mesh Ewald algorithm, and the non-bonding interaction intercept was set as 1.2 nm (Hou et al., 2023). As part of the post-simulation analysis, we verified whether the conformations of the complexes generated throughout the 100-ns interval were stable and showed any variations in the binding and atomic structure. Thus, the initial conformations of the complexes after minimisation and NPT/NVT equilibration were retrieved and superimposed with the structures obtained from the simulations at regular intervals of 10 ns. Finally, all 11 frames were collected and superimposed to check for conformational changes over the simulation period. These structural ensembles were also used to analyse the conservation of the amino acid residues involved in the intermolecular interactions between the NGFs and TrkA.

### 2.8 Molecular mechanics Poisson–Boltzmann surface area (mmPBSA) analysis

The binding affinities of the protein–protein complexes were analysed using their mmPBSA values (Miller et al., 2012). In this study, the g_mmpbsa tool of GROMACS was employed to calculate the binding affinities of the simulated NGF-TrkA complexes for the full 100 ns molecular dynamics trajectory at regular intervals (Kumari et al., 2014). The binding free energies were then decomposed into the van der Waals, electrostatic, solvation, and entropy components for 10,000 frames using the MMPBSA.py script. The total binding energy (ΔG_binding_) of the complex in a solvent is defined as

$$\Delta G_{\text{binding}=}\;G_{\text{complex}}-\left(G_{\text{receptor}}+G_{\text{protein}}\;\right)$$

where G_complex_ is the total energy of the receptor–protein complex; G_receptor_ and G_protein_ are the individual energies of the respective protein molecules. The final ΔG_binding_ values for the receptor–protein complexes are the average values from the molecular dynamics simulation trajectories over the 100 ns interval (Kollman et al., 2000). Here, block averaging was used to estimate the statistical errors.

### 2.9 Principal component analysis (PCA)

We employed PCA to verify the conformational dynamics of drNGF-TrkA, nnNGF-TrkA, hNGF-TrkA, drNGF, nnNGF, and TrkA. PCA is also known as essential dynamics and helps in understanding the overall motions of the proteins via analyses of the correlated atomic movements and trajectories to identify the dominant movements of the system. The covariance matrix of these movements was first computed using the C-α backbone in GROMACS via the g_covar tool; then, the matrix was diagonalised to obtain the eigenvectors representing the directions of the movements and eigenvalues signifying the amplitudes of the movements. These values describe the collective dynamics of the protein–protein complexes, as reported previously (Borkotoky and Murali, 2017). This dimensionality reduction technique allows construction of a covariance matrix of atomic displacements and reveals the principal components representing the most significant and coordinated motions. The majority of the dynamic behaviours of the system are adequately captured by the first few principal components, making it easier to analyse the significant conformational changes. The cosine content (ci) of each principal component of the covariance matrix was computed to produce the free energy landscape (FEL); the gmx_sham script was used to create the FEL plot.

## 3 Results

### 3.1 Multiple sequence alignment (MSA)

**FIGURE 1:**
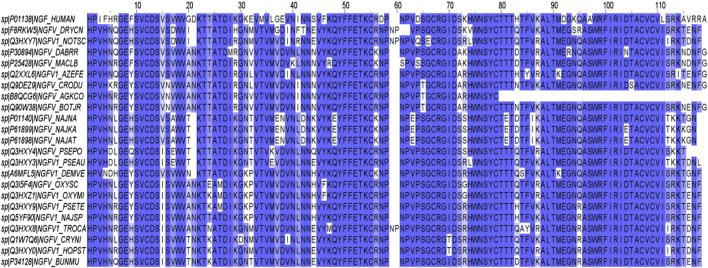
Multiple sequence alignment of nerve growth factors (NGFs) from humans and various species of snakes.

There are a total of 112 sNGF sequences deposited in the UniProt database belonging to 71 different snake species, of which only 38 have been reviewed. Only the mature sNGF sequences reviewed in UniProt were considered in our MSA analysis, and the results of the MSA are shown in Figure 1 using Jalview software (Waterhouse et al., 2009); here, the residues marked with an asterisk (*) are conserved (11.96%) throughout the NGF family and provide information about the NGF signature sequence. Although the number of sNGF sequences reviewed in the database was 38, only 23 were considered in our MSA analysis because some of these were isoforms of the same protein and were thus excluded. The pairwise alignment result for each sNGF with hNGF was obtained, and these results helped us to narrow down the species sources of the NGFs that were highly similar to hNGF (Supplementary Table S1). The physicochemical characteristics of the sNGFs were predicted using various tools, and the results support the idea that the properties of these sNGFs are similar to those of hNGF (Supplementary Table S2).

### 3.2 Structural characteristics of the NGFs

**FIGURE 2:**
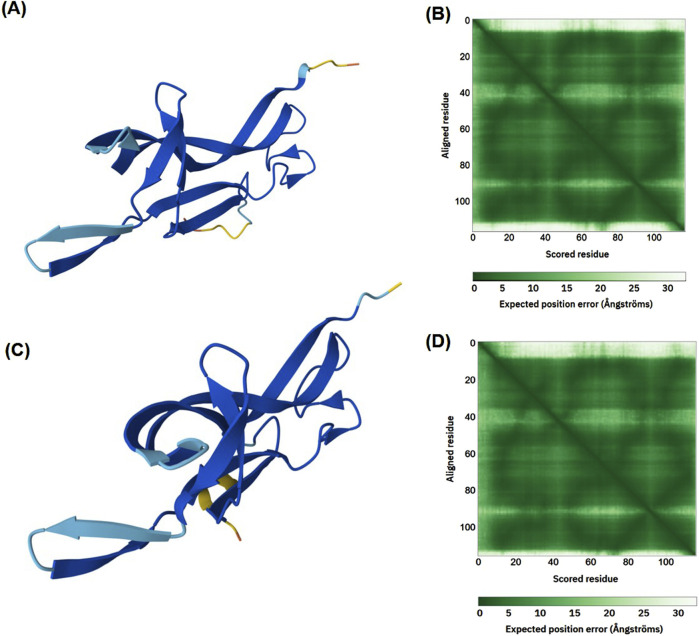
Structures of **(A)** drNGF and **(C)** nnNGF predicted by AlphaFold2; the confidence levels are indicated in blue (high confidence, pLDDT > 90) and cyan (moderate confidence, 70 < pLDDT < 90). Predicted aligned error scores of **(B)** drNGF and **(D)** nnNGF shown as colour variations, where the green regions signify well-modelled structures with high confidence and minimal errors.

The signature of the NGF family is given by the amino acids at positions 64–77 (GCRGIDAKHWNSYCT), as found using the InterPro tool. The secondary structures of drNGF, nnNGF, and hNGF are similar, consisting of β-sheets (53%–59%) and additional α-helices (0%–4.5%) only in nnNGF. The secondary structure of NGF consists of two β-strands that are connected together by loops. In all three species, there are higher proportions of hydrophilic amino acids in the β-sheets (24.71%) and loops (26.72%), while there are higher proportions of hydrophobic amino acids in the α-helices (0.86%). The 3D structures of drNGF and nnNGF predicted using the AlphaFold2 tool, along with the quality metrics like pLDDT and PAE, are shown in Figures 2A–D. The NGF structures predicted for *D. russelii* and *N. naja* using AlphaFold v2.0 and v3.0 are similar, with RMSD values of 0.353 Å and 0.363 Å, respectively; the predicted structures are also close to the experimentally derived structure of NGF from *Naja atra*, for which the Ramachandran plots show conformations of 94.39% and 95.28%, respectively, indicating that all the residues are positioned in energetically favourable regions.

### 3.3 Correlations with hNGF

The sNGF sequences from the various species were obtained and aligned in a pairwise manner with hNGF to identify species with the highest match that were also predominantly found in the Indian subcontinent. The similarity and identity indexes of the sNGFs with hNGF are given in Table 1. Superposition of the structures of drNGF and nnNGF with hNGF (1WWW) revealed RMSD values of 0.747 Å and 0.655 Å, respectively, when visualised using PyMol (Schrödinger, n.d.). Here, the deviations were found to be highest around the N-terminus and at the C-terminus.

**TABLE 1:** Identities and similarity indexes of drNGF and nnNGF with hNGF. The values in the upper diagonal represent the percentage identities, while those in the lower diagonal represent the percentage similarities.

Identity/ Similarity	hNGF	drNGF	nnNGF
hNGF	100	70.9	67.5
drNGF	87.2	100	75.6
nnNGF	80.3	84.9	100

### 3.4 Coevolutionary analysis

The amino acid residue pairs that played critical roles in maintaining the structures and functions of the proteins as well as those that were highly sensitive to mutations were identified from their evolutionary couplings with the other residues derived from MSA analysis. The EVcouplings web server was used to predict the coevolving residues in drNGF, nnNGF, and hNGF, and the results show that 85% of the predicted contacts are correlated with the 3D structure. These predicted contacts are likely important for maintaining the structural fold and receptor-binding capabilities, as shown in Supplementary Figure S1. A high evolutionary score indicates these residues are indeed in contact within the 3D structure; thus, these 85% predicted residues were considered to be highly conserved with coevolved interactions, while the remaining 15% predicted contacts were deemed to be low-conserved interacting pairs. Covarying residues are those that have been examined in the course of the evolution. Given this information, coevolutionary analysis is an effective method for maintaining the structure and function of a protein (Wang et al., 2020; Zhang et al., 2022).

The conserved residue pairs were selected on the basis of the following criteria while maintaining the default parameters. Initially, the conserved amino acid residues of the proteins were mapped through MSA of all the sNGFs. Then, the amino acids that remained identical throughout all species were taken as the conserved residues, which included up to 41 amino acids out of 116 on average (35.34%). The amino acid pairs in the sequences were next identified using EVcouplings, and the conserved amino acid pairs were selected for further analyses, as shown in Table 2. After mapping the conserved amino acid pairs (coevolutionary coupled amino acids), substitution analysis was performed; if there were any substitutions in the conserved amino acids of the coevolved residue pair, then the other amino acids of the pair would also mutate to maintain the same types of bonding, fold, and structure. The program searches for the most compatible amino acids based on evolutionary trends at the paired locations and also checks for energetically favourable couplings. The substituted amino acid pairs were also found to be conserved across drNGF, nnNGF, and hNGF herein, as shown in Table 2.

**TABLE 2:** Conserved amino acid pairs observed in the three species of NGFs (drNGF, nnNGF, and hNGF) and analyses of their substitutions.

Original and conserved couplings in the NGFs	Substituted couplings
E8, T78	Y8, Q78
C12, V35	A12, C35
W18, Y49	V18, K49
A25, V35	S25, D35
D27, R97	A27, V97
F50, A86	V50, I86
W73, N74	G73, T74
T78, V108	A78, T108
A86, I99	R97, T88
L87, W96	D87, T96
T88, I99	V88, R99
I99, I101	S99, D101
R100, I101	S100, I101
A104, V106	W104, I106

**FIGURE 3:**
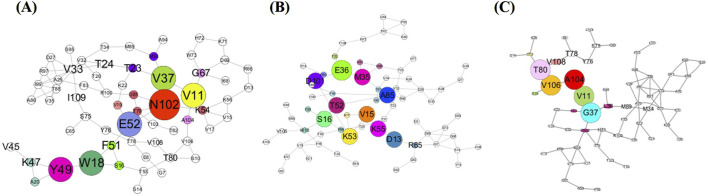
Residue coevolution networks of **(A)** drNGF; **(B)** nnNGF; **(C)** hNGF highlighting the key hub residues involved in the structural and functional connectivities.

To visualise the evolutionary coupling networks, we performed network analysis on the EVcouplings from the server. In humans, there is a centralised core network with V106, A104, T80, V11, and G37 as the principal hub residues, displaying a clear hierarchical organisation with these residues mediating most interdomain organisation (Figure 3A). In drNGF, there is a more dispersed bug pattern with N102, E52, V37, Y49, W18, and V11 as the key residues; this pattern shows many interaction clusters rather than a centralised hub like hNGF. In nnNGF, there is a centralised core formed by the residues E36, M35, D101, T52, V15, S16, K53, K55, D13, and A85 with substantial branching connections throughout the network. While nnNGF has more charged residues as hubs (E36, D101, D13) than hNGF, V11 seems to be a key residue in both hNGF and drNGF, indicating the importance and conservation of hydrophobic interactions for structural relevance (Figure 3).

### 3.5 HotSpot analysis

Herein, hotspots refer to the amino acid residues that contribute to the highest or most pronounced binding free energy (ΔΔG_binding_) ≥ 2.0 kcal/mol in a protein–protein complex (Moreira et al., 2007). The binding energy difference indicates the difference in free energies between the mutant and wild type given by

ΔΔG=ΔGMUTcomplex−ΔGWTcomplex.

The amino acid residues of NGF that were considered as hotspots were H4, E11, I71, D72, H84, W99, R103, and V111, whereas the hotspot residues for TrkA were E295, H297, R347, N349, and Q350 (Krüger and Gohlke, 2010; Sukhwal and Sowdhamini, 2013; Melo et al., 2016).

### 3.6 Molecular docking

**FIGURE 4:**
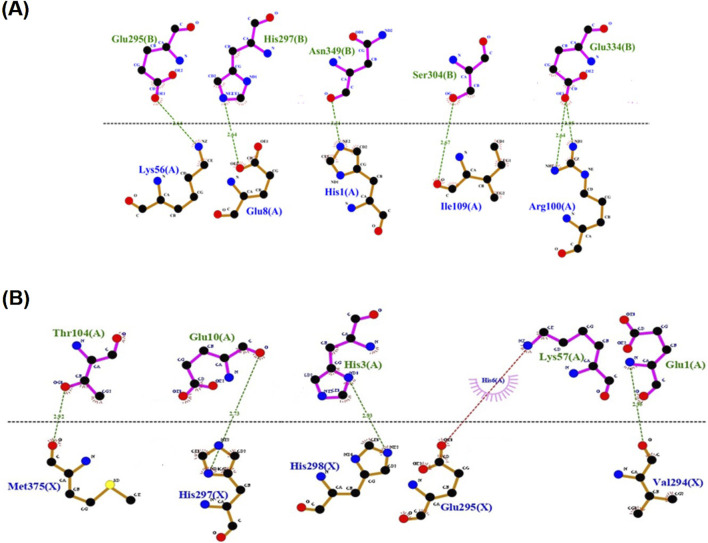
Two-dimensional representations of the docked complexes of **(A)** drNGF-TrkA and **(B)** nnNGF-TrkA visualised using Dimplot of Ligplot; the docked complexes were obtained from HADDOCK. The hydrogen bonds are shown in green while the red dotted lines indicate salt bridges.

Out of the 1,000 complexes generated, 100–400 were clustered and only around 40 models were obtained from HADDOCK as having the lowest energy conformations based on the RMSDs from the overall energy structure and individual energy components (van der Waals, electrostatic, desolvation, restraints violation, and hydrophobic energies as well as buried surface areas and Z-score values) (https://rascar.science.uu.nl/haddock2.4/). To ascertain the similarities of the binding modes between hNGF-TrkA and sNGF-TrkA, it is necessary to confirm maintenance of the binding residues as well, which can be achieved by correlating the sequences with hNGF as a prerequisite for subsequent work. Initially, the binding interface for the hNGF-TrkA complex was identified from the PDB file (1WWW) and correlated with that of the sNGF-TrkA complex; here, the active site residues were H4, E11, W21, R59, H84, and R103 in hNGF, and the corresponding residues in sNGF were treated as the interacting sites for docking. We did not perform blind docking but instead used residues reported in literature as the restraints. The complex that had the best score, lowest binding energy, and highest correlation with the binding site residues of hNGF was selected (Dominguez et al., 2003; Van Zundert et al., 2016; Kozakov et al., 2017). This complex was visualised for the 2D interactions between the proteins of drNGF, nnNGF, and TrkA using LigPlot+ v.2.2.8 (Wallace et al., 1995; Laskowski and Swindells, 2011), as shown in Figures 4A,B. By aligning the residues of the hNGF-TrkA binding interface with the equivalent residues of sNGF-TrkA, we noted over 50% conservation for drNGF-TrkA and up to 62.5% conservation for nnNGF-TrkA, as given in Table 3. Given the conservation of the interacting amino acids in the sNGF sequences, it is possible to mimic and construct the complex model of sNGF-TrkA using the crystal structure of hNGF-TrkA as the template (Wiesmann et al., 1999).

**TABLE 3:** Interacting residues in NGF-TrkA. The interacting residues for hNGF-TrkA were obtained from literature, while those for drNGF and nnNGF were correlated to hNGF (Devi and Jayaraman, 2025).

hNGF, TrkA	drNGF, TrkA	nnNGF, TrkA
H4, F303	H1, F303	H3, F303
R9, E334	Q6, E334	L8, H297
E11, H297	E8, H297	E10, H297
E11, R347	E8, R347	E10, R347
W21, H353	W18, H353	W20, H353
R59, E295	K56, E295	K57, E295
H84, Q350	D81, Q350	D82, Q350
R103, N349	R100, N349	R101, N349

Initial docking and simulation studies were performed on the docked complexes from the HADDOCK and ClusPro tools. However, HADDOCK was preferable since its predicted intermolecular interactions were consistent with the residues reported in literature. The complexes generated by HADDOCK converged during simulations and exhibited lower RMSD values. In contrast, the complexes generated by ClusPro had higher RMSD values (0.8–0.9 Å) during the simulations, so the final analysis was performed using the HADDOCK models. Statistical analyses of the docking results show that the interacting residues S14 and E295 have maximum occurrences in drNGF-TrkA, while residues S16 and E295 have the maximum hits in nnNGF-TrkA. When correlating the binding site residues with findings reported in literature, our data showed that overlaps with interacting residues K56 and E295 for drNGF-TrkA as well as E10 and E347 for nnNGF-TrkA had the maximum occurrences, which support the reliability of our docking results (Supplementary Figures S2A and S2B).

### 3.7 Molecular dynamics simulation

**FIGURE 5:**
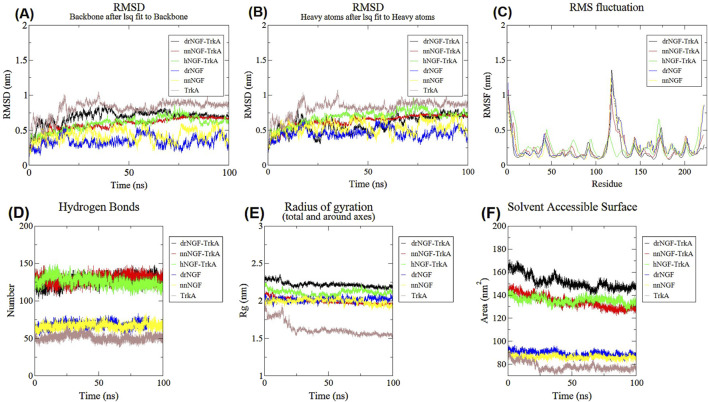
Molecular dynamics simulation results of drNGF-TrkA, nnNGF-TrkA, hNGF-TrkA, drNGF, nnNGF, hNGF, and TrkA. **(A)** Graph showing the root mean-squared distance (RMSD) values of the backbone structures of all the complexes; **(B)** RMSD of the side chains; **(C)** root mean-squared fluctuation (RMSF) values; **(D)** numbers of hydrogen bonds in the different complexes; **(E)** radius of gyration; **(F)** solvent accessible surfaces of the complexes.

Out of the many docked complexes generated herein, only one complex was considered for further simulation studies based on its similarity and maximum interactions, as given in literature. Six systems were simulated individually (drNGF with and without TrkA, nnNGF with and without TrkA, hNGF with TrkA, and TrkA alone) for 100 ns each. The trajectories were analysed for stability, with all the complexes being checked in their monomeric states. If the ligand–receptor complexes are more stable than the individual ligands (sNGF and hNGF), we can conclude that complex formation is essential for stability; herein, the complex forms showed greater stability than the ligands alone, suggesting that the interactions between the ligands and receptors play key roles in maintaining stability. All of the post-simulation analysis results were plotted using QTGRACE software. The molecular dynamic simulations were performed thrice for the *D. russelii* and *N. naja* species using different starting structures and different geometries to better capture the binding variabilities. The structural deviations in the molecular structures of the complexes were analysed by calculating the RMSDs of the backbone and side chain. Differences in the RMSD values provide insights into the overall stability of the backbone of the protein in the apo and complexed forms. Lower RMSD changes suggest higher stability since there are fewer deviations, indicating convergence of the structures. The drNGF-TrkA complex attained stability at approximately 75 ns and remained steady thereafter with an RMSD of 0.72 nm. When drNGF alone was simulated, it showed major deviations from the average RMSD value of 0.34 nm and stabilised at approximately 65 ns, while the RMSD values varied between 0.45 and 0.6 nm (Figure 5A) (Hou et al., 2023; Karunakaran and Muniyan, 2023).

The nnNGF-TrkA complex stabilised at approximately 30 ns with an average RMSD between 0.5 and 0.6 nm, while nnNGF exhibited an average RMSD value between 0.2 and 0.5 nm, as shown in Figure 5A. The reference used throughout the study was hNGF-TrkA, which also stabilised at approximately 25 ns and maintained an average RMSD of 0.59 nm with minor fluctuations at 50 ns and 72–74 ns. When TrkA was simulated, the RMSD values stabilised around 35 ns and remained in the range between 0.75 and 0.85 nm throughout the simulation, with a minor spike at 90 ns showing an RMSD of 0.91 nm. Since the structure of the complex converged and was stable for the last 50 ns of the simulation period, molecular dynamics simulation was not performed for a longer period of time. The average RMSD values during the 50 ns durations were 0.585 ± 0.12 nm, 0.583 ± 0.101 nm, and 0.592 ± 0.103 nm for the drNGF-TrkA, nnNGF-TrkA, and hNGF-TrkA complexes, respectively. Since the standard deviations for all the complexes were 0.1, there is not much variability in the RMSD values after 50 ns, which confirms stability of the trajectory and hence the adequacy of a 100 ns simulation. Many studies have been published in recent times with 100-ns simulations that have displayed stable structural convergences (Resende-Lara et al., 2020; Rosilan et al., 2024; Paul et al., 2025). The average RMSD of the heavy atoms of the amino acids of drNGF-TrkA was 0.59 nm, which reached stability at approximately 50 ns with an RMSD between 0.5 and 0.7 nm for the remaining simulation period (Figure 5B). The nnNGF-TrkA system reached stability at approximately 30 ns and had almost constant RMSD values between 0.5 and 0.6 nm over the entire simulation period. The hNGF-TrkA complex reached stability at approximately 25 ns and maintained an average RMSD of 0.68 nm throughout. Although TrkA had a relatively higher RMSD of 0.82 nm than drNGF and nnNGF, it did not stabilise upon simulation and may thus not be stable in a biological system (Figure 5B).

The root mean-squared fluctuation (RMSF) determines the flexibility of the individual amino acids of a protein through the average manoeuvrability of the C-α atoms. Unstructured loops usually result in higher RMSF values, while α-helices and β-sheets have lower values. The amino acid residues of the sNGFs binding to TrkA are stable and even showed lower RMSF values than the corresponding hNGF residues. The average RMSF values of the active site residues of drNGF, nnNGF, and hNGF when bound to TrkA are 0.28 nm, 0.20 nm, and 0.35 nm, respectively; however, the calculated RMSF values of drNGF and nnNGF in the apo form are 0.35 nm and 0.27 nm, respectively. Since the active site RMSF of NGF in the bound form is less than that in the apo form, the bound form of NGF-TrkA is implied to fluctuate less than the apo form, as shown in Figure 5C and Supplementary Table S3. The active site residues show fluctuations of approximately 2.0 nm, suggesting the high stability of the complex, with 0.3 nm being the overall fluctuation for all complexes. This means that the complexes exhibit strong stabilisation as the active sites and their interacting residues remain stable with minimal fluctuations (Karunakaran and Muniyan, 2023).

Hydrogen bonds are crucial for the stability and folding of the protein complex, and the number of bonds at the binding interface remains constant. The average numbers of hydrogen bonds in the interfaces between drNGF and TrkA, nnNGF and TrkA, and hNGF and TrkA are 126, 118, and 126, respectively. The average numbers of hydrogen bonds formed in drNGF, nnNGF, and TrkA are 68, 66, and 51, respectively (Figure 5D). The radius of gyration (Rg) quantifies the RMSD of each atom from the central axis, which is crucial for the structural properties of protein complexes like compactness, stiffness, and folding; lower Rg values indicate more stability. The Rg values of the drNGF-TrkA, nnNGF-TrkA, and hNGF-TrkA complexes remained stable at 2.22 nm, 2.23 nm, and 2.11 nm, respectively, over the 100-ns simulation interval, as shown in Figure 5E. The drNGF, nnNGF, and TrkA structures had Rg values of approximately 2.02 nm, 1.99 nm, and 1.63 nm, respectively (Karunakaran and Muniyan, 2023). The solvent accessibility surface area (SASA) is a measure of the accessible surface area of a protein molecule to water; a higher SASA value suggests that a complex is more accessible to external molecules, while a lower SASA value indicates limited accessibility. The average SASA values in this study were 128.64 nm^2^ for drNGF-TrkA, 130.07 nm^2^ for nnNGF-TrkA, and 117.20 nm^2^ for hNGF-TrkA (Figure 5F). For the individual proteins, the SASA values were 67.01 nm^2^ for drNGF, 65.96 nm^2^ for nnNGF, and 78.155 nm^2^ for TrkA. From these findings, it is evident that the complexes had attained proper structural conformations during the molecular dynamics simulations (Karunakaran and Muniyan, 2023).

It can be seen from the statistical analysis that the RMSD of the main chain, RMSDs of the side chains, RMSF values, Rg, SASA values, and number of H-bonds of the sNGFs are similar to those of the reference hNGF-TrkA complex; thus, we can use the sNGF-TrkA complexes as alternatives to the native complex with hNGF. The average values of all the metrics from the molecular dynamics simulations show *p*-values < 0.001 when plotted using Numpy and prove their statistical significances (as summarised in Supplementary Table S4). Statistical analyses of the simulated complexes showed that the RMSD values of the drNGF-TrkA and nnNGF-TrkA complexes indicated better stabilities and lower deviations in the structural characteristics than those of the hNGF-TrkA complex. The fluctuations of the individual amino acids of the sNGF-TrkA complexes also showed lower values than those of the hNGF-TrkA complex, indicating more rigidity. As the number of amino acids in a protein complex increases, its surface area for solvent accessibility and number of hydrogen bonds also increase statistically, as shown in Supplementary Figure S3.

**FIGURE 6:**
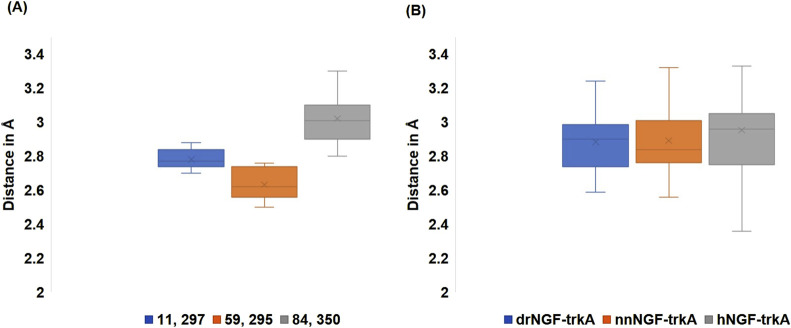
**(A)** Lengths of the hydrogen bonds formed between the active site residues that were maintained throughout the simulation period. The active site residues (E11, H297), (R59, E295), and (H84, Q350) are shown in blue, orange, and grey colours, respectively. **(B)** Lengths of the hydrogen bonds formed between all the interacting site residues over the entire simulation period of 100 ns; drNGF-TrkA, nnNGF-TrkA, and hNGF-TrkA are represented in blue, orange, and grey colours, respectively.

The initial conformations of the docked complexes after energy minimisation and equilibration were obtained and superimposed with different conformations obtained at regular time intervals of 10 ns throughout the simulation period of 100 ns. When considering the hydrogen bonds between all types of interacting amino acids, we noted that these bonds were formed at distances of 2.6–3.4 Å in drNGF-TrkA, 2.6–3.3 Å in nnNGF-TrkA, and 2.4–3.82 Å in hNGF-TrkA, as shown in Figure 6A. The interacting residue pairs that were considered as active site residues were conserved and maintained throughout the simulation period; these included E11 and H297 for drNGF-TrkA, R59 and E295 for nnNGF-TrkA, and H84 and Q350 for hNGF-TrkA. Among these three complexes, nnNGF-TrkA exhibited R59 and E295 interactions consistently; the strongest hydrogen bonding here suggests stronger and more stable interactions. The H84 and Q350 interactions in hNGF-TrkA exhibited the largest variations and highest average distance, indicating relatively weaker interactions. The distances of the hydrogen bonds between these interacting pairs were checked throughout the simulation period of 100 ns and are plotted in Figure 6B. It can be seen that all three NGF-TrkA complexes maintain stable hydrogen bond lengths with average distances below 3 Å, suggesting consistent interactions with no significant weakening or disruptions over the simulation period.

**FIGURE 7:**
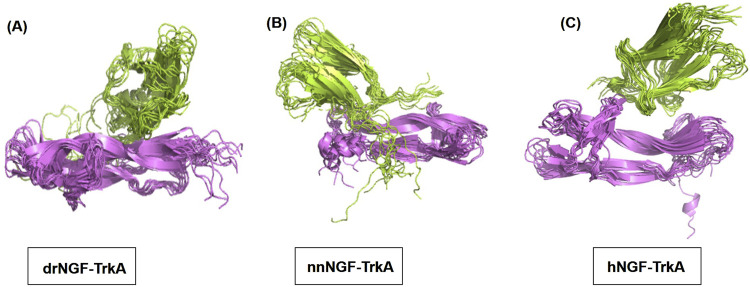
Structures obtained by superimposing the 11 conformers obtained throughout the simulation period at intervals of every 10 ns, with 1 ns being the reference starting point. The NGFs are shown in violet colour, while TrkA is shown in green colour. **(A–C)** drNGF-TrkA, nnNGF-TrkA, and hNGF-TrkA structures, respectively.

**FIGURE 8:**
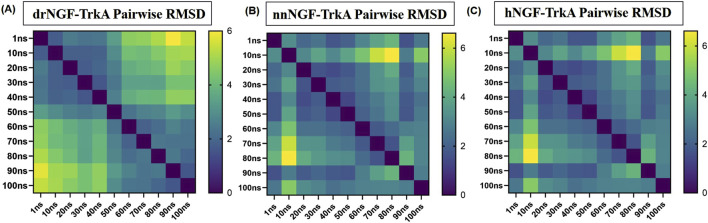
Pairwise RMSD values of the three complexes, where drNGF-TrkA shows the highest variation initially but gradually reduces and stabilises over time. Overall, the average pairwise RMSD values are similar for **(A)** drNGF-TrkA; **(B)** nnNGF-TrkA; **(C)** hNGF-TrkA, indicating that they are conserved. Low RMSD values indicate similar conformational states, while higher RMSD values indicate a range of conformational states. The RMSD values are low towards the higher end of the time scale of the molecular dynamics simulations, indicating stable structures of the NGF-TrkA complexes.

The RMSD values were obtained by superimposing the structures at every 10 ns by considering the 1 ns structure as reference; this yielded 0.838 Å for drNGF-TrkA, 0.987 Å for nnNGF-TrkA, and 1.029 Å for hNGF-TrkA, as shown in Figure 7, indicating that the structural conformations are almost similar for all three complexes without much variations throughout the simulation period. The pairwise RMSD values were generated by considering the 1 ns structure as the reference and the conformers at steps of 10 ns over the simulation period to generate the graphs shown in Figure 8. The average RMSD values generated from the pairwise interactions are 3.23 Å for drNGF-TrkA and 2.88 Å each for both nnNGF-TrkA and hNGF-TrkA. The darker colours in the figure represent higher RMSD values, where each matrix element represents the RMSD between the structures at the corresponding time points shown along the x- and y-axes. Low RMSD values indicate similar conformational states, while higher RMSD values indicate a range of conformational states. Comparison of the RMSD shows lower values at higher points in the time scale of the molecular dynamics simulation, indicating stable structures of the NGF-TrkA complexes at these points. The best pairwise RMSD values were observed for nnNGF-TrkA as compared to hNGF-TrkA and drNGF-TrkA.

### 3.8 Prediction of binding energy, binding affinity, and dissociation constant

The binding affinity is expressed in terms of either the Gibbs free energy (ΔG, kJ/mol) or the dissociation constant (K_d_, M) calculated using PRODIGY. The interfacial contacts (ICs) are the amino acid residues that interact with the residues of other proteins within a well-defined cutoff distance of 5.5 Å, while the non-interacting surfaces (NISs) indicate the parts that do not interact. As the number of ICs increases, the binding strength also increases as the interfaces are more tightly bound owing to the higher interaction strength. Of the three docked complexes, nnNGF-TrkA exhibited the highest binding energy (−51.04 kJ/mol), indicating better interactions between the interactors (nnNGF and TrkA); the number of ICs in nnNGF-TrkA is 89, which was the highest among the three complexes, while the number of ICs in drNGF is 83, as shown in Table 4. The NISs are also known to allosterically affect the interacting contacts (Honorato et al., 2021).

**TABLE 4:** PRODIGY results of drNGF, nnNGF, and hNGF; the higher the Gibbs free energy value, the more stable is the complex. Here, nnNGF and drNGF both have higher Gibbs free energies than hNGF, indicating the greater stability of sNGFs. IC: interfacial contact; NIS: non-interacting surface.

Parameter	drNGF	nnNGF	hNGF
ΔG (kJ/mol)	−45.60	−51.04	−30.54
K_d_ (M) at 25 °C	1.1 × 10^−8^	1.1 × 10^−9^	4.7 × 10^−6^
ICs charged–charged	9	7	0
ICs charged–polar	12	17	6
ICs charged–apolar	24	22	10
ICs polar–polar	2	5	2
ICs polar–apolar	11	22	6
ICs apolar–apolar	25	16	2
NIS charged	16.16	18.69	18.32
NIS apolar	43.94	42.93	43.46

Post-simulation mmPBSA analysis showed that the drNGF-TrkA and nnNGF-TrkA complexes achieved more favourable binding energies than hNGF-TrkA. The results of the mmPBSA analysis suggest that drNGF-TrkA and nnNGF-TrkA have higher binding energies, meaning that drNGF and nnNGF have more affinity towards TrkA than hNGF, as presented in Table 5. The strong binding energies predicted for the sNGFs could imply higher potency and efficacy of the protein function with improved selectivity as well as ability to stay bound to the receptor for a longer period of time; this high binding affinity may be attributed to the differences in the decomposition of each of the residues in the protein toward the protein interactions. The major energy components contributing to the binding energies of the molecules are van der Waals energy, electrostatic interactions, and polar solvation energy for drNGF-TrkA; van der Waals energy, electrostatic interactions from the Poisson–Boltzmann calculations, and polar solvation energy for nnNGF-TrkA; as well as van der Waals energy and electrostatic interactions for hNGF-TrkA. Altogether, the van der Waals and electrostatic interactions play pivotal roles and contribute significantly to the binding energies of all three complexes.

**TABLE 5:** Molecular mechanics Poisson–Boltzmann surface area analysis of the binding affinities of different NGFs with TrkA.

System	ΔG binding energy (kcal/mol)	Electrostatic (kcal/mol)	van der Waals (kcal/mol)	Polar solvation (kcal/mol)
drNGF-TrkA	−65.55 ± 13.96	−305.22 ± 43.55	−88.13 ± 10.16	339.73 ± 44.75
nnNGF-TrkA	−58.59 ± 19.94	44.97 ± 41.87	−80.83 ± 12.04	−10.49 ± 42.14
hNGF-TrkA	−28.27 ± 10.08	−308.89 ± 47.90	−36.75 ± 7.11	323.59 ± 45.44

The residues that contribute favourably to the interaction between TrkA and drNGF are P2, V3, H4, S10, V11, F51, T78, T80, R100, A104, C105, V106, V108, and I109, while D81 is unfavourable to the interaction; although many residues contributed favourably to the interactions with TrkA, R100 and T80 showed the highest affinities with the least energies. There are various other residues excluding those noted above that contribute to the binding energy; these are either conserved (80%) or similar in species like *Naja* and hNGF but do not appear to cause any significant changes. The amino acids H4, D81, and I109 are conserved in the sNGFs but are replaced by F, H, and L in the case of hNGF, respectively; these differences in the amino acids contributing to the energy values could be responsible for the enhanced binding affinities of sNGFs compared to that of hNGF. Although the numbers of amino acids contributing to the NGFs in the three species are similar, the number of amino acids significantly contributing to the energy required to bind to TrkA vary considerably from 15 in *D. russelii* to 16 in *N. naja* and only four in hNGF. Hence, there are a large number of amino acids that do not contribute significantly to the binding affinity with TrkA, which could be one of the reasons for the reduced binding affinity of hNGF compared to the sNGFs. As the number of amino acids contributing significantly and favourably to the binding increases, the greater is the binding affinity.

### 3.9 PCA

By analysing the eigenvectors, we can understand the general motions of a protein as a sum of its individual atomic movements, which are indicative of its shape changes and biological roles. The eigenvector is a linear combination of the atomic movements depicting the overall motions of the protein; these atomic movements represent the deformation modes and biological activities (Pant et al., 2020). Therefore, PCA was performed to identify the eigenvectors responsible for the global movements of the proteins to ensure stability of the complexes. Since the global movements of the proteins are primarily dictated by the first few eigenvectors, we diagonalised the covariance matrices and selected the first two principal components, which accounted for approximately 80% of the total motions of the proteins. Generally, a lower eigenvalue with a higher eigenvector indicates a less dynamic and more stable system. The percentage concentrations of the first 10 eigenvectors of each complex were examined to understand their motion characteristics. Although hNGF-TrkA exhibited the highest stability with the selected contributions accounting for 94.37% of the total conformation, the sNGFs also exhibited considerably stable conformations of 79.52% and 72.73% for nnNGF-TrkA and drNGF-TrkA, respectively. Furthermore, the apo forms of the complexes contributed up to 78.58%, 76.257%, and 70.47% of the stability via drNGF, nnNGF, and TrkA, respectively.

**FIGURE 9:**
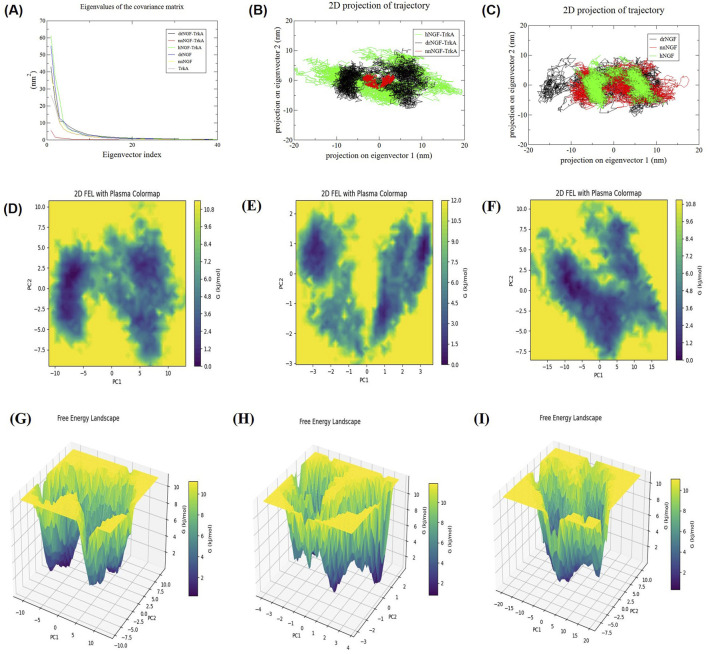
**(A)** Eigenvalues of the covariance matrices of different molecular systems, showing the variance captured by each eigenvector. **(B)** 2D projections of the molecular trajectories onto the first two principal eigenvectors for drNGF-TrkA (black), nnNGF-TrkA (red), and hNGF-TrkA (green). **(C)** 2D trajectory projections for drNGF (black), nnNGF (red), and TrkA (green), highlighting their spatial distribution and overlap. **(D–F)** Two-dimensional free energy landscape (FEL) plots of drNGF-TrkA, nnNGF-TrkA, and hNGF-TrkA, respectively. The basins correspond to stable conformations, where the basin depths indicate the relative stabilities in terms of the energy minima of the biomolecules and the shapes indicate the flexibility and diversity of the probable conformational states. The crest regions of the plots indicate unstable or transient conformations. **(G–I)** Three-dimensional FELs of drNGF-TrkA, nnNGF-TrkA, and hNGF-TrkA, respectively.

As shown in Figure 9A, the nnNGF-TrkA complex alone is similar to and has a slightly higher value than hNGF-TrkA in terms of the eigenvalues and eigenvector, indicating that nnNGF-TrkA is just as stable as hNGF-TrkA save for some minor fluctuations; here, the hNGF-TrkA complex serves as a reference with a tightly constrained conformational distribution indicative of highly refined and energetically favourable interactions. The eigenvalue plot (Figure 9A) illustrates the rapid decay of the eigenvalues across all systems, indicating that only a few principal components govern the overall motions. This trend suggests that the major conformational changes in the sNGF-TrkA complexes are constrained to a limited subspace, reinforcing their structural stabilities. As far as PCA is concerned, lower conformational spaces indicate higher stabilities of the protein–protein complex systems. The 2D projections of the principal components of the NGF-TrkA complexes display more compact distributions than their apo forms, indicating that complex formation is required and mandatory for the stability of the protein complex.

According to our findings, hNGF spans a broader conformational space than the sNGF variants, suggesting greater structural flexibility. The extended sampling in hNGF-TrkA might indicate its wider range of functional states or dynamic adaptability. Although the drNGF-TrkA system displays a slightly broader distribution than the nnNGF-TrkA complex that could indicate some degree of conformational flexibility, it exhibits a more constrained dynamic signature than hNGF and suggests greater rigidity. However, the nnNGF-TrkA complex exhibits more restricted motions and a well-defined conformational space, which reinforce its structural stability as well as robust and stable binding modes (Figure 9B). The restricted conformational spaces exhibited by the sNGFs may indicate stronger or sustained TrkA activation with fewer conformational states, along with stable protein–protein interactions (Resende-Lara et al., 2020). Subtle changes in the conformational states or structures of the NGFs could impact their ability to activate TrkA (Resende-Lara et al., 2020). Despite the evolutionary differences among the complexes, their overlaps in the core area may indicate shared structural characteristics and perhaps conserved binding mechanisms. The unique signature regions of the individual complexes suggest specific adaptations that may be correlated with their distinct biological activities.

Notably, there are differences in the dynamics of the individual NGF proteins, with drNGF displaying greater flexibility than nnNGF that exhibits a more rigid structure (Figure 9C). Furthermore, TrkA alone exhibited the highest degree of flexibility, confirming its inherent dynamic nature in the unbound state. Upon binding with the different NGFs, we observed reductions in the conformational spaces across all variants, albeit to varying extents, highlighting the stabilising effects of ligand binding (Figure 9C). The sNGFs may have evolved towards more constrained conformational dynamics when bound to TrkA than hNGF. These insights are not only valuable for advancing our fundamental understanding of the biology of neurotrophins but also hold significant implications for the development of NGF-based therapeutic strategies.

We generated FEL plots for drNGF-TrkA, nnNGF-TrkA, and hNGF-TrkA over the 100-ns simulation intervals; these plots describe the minimum energy conformations of the protein–protein complexes. Complexes with a greater stability achieve unique conformations with stronger energy and minimal energy clusters, while complexes with multiple minimum energy clusters and weak interactions indicate an ensemble of possible conformers (Figure 9). The FEL of a biomolecule represents its different energy states during simulation. The areas with darker patches indicate the conformational spaces of the biomolecules; plots with fewer and well-defined patches indicate restricted conformational changes and greater stability for the corresponding biomolecules, while broader and scattered patches indicate wider conformational sampling indicative of flexibility. The FEL plot of nnNGF-TrkA (Figure 9E) is well-defined and indicates stable conformations, although there could be subtle differences in the energy. However, the FEL plots of hNGF-TrkA and drNGF-TrkA are broader, suggesting that these complexes are relatively flexible and energetically unstable (Figures 9D,F).

The 3D FEL plots show broad basins and high-energy minima with lower occurrence of deeper basins in drNGF-TrkA (Figure 9G), whereas there are deeper basins but low-energy minima in nnNGF-TrkA (Figure 9H). The 3D plot of hNGF-TrkA displays a single broad basin with high-energy minima (Figure 9I); this indicates that hNGF-TrkA and drNGF-TrkA may have greater conformational freedom than nnNGF-TrkA. Hence, drNGF-TrkA and hNGF-TrkA complexes have shallow landscapes with more fluctuating and metastable states, while nnNGF-TrkA has a deeper basin suggesting its thermodynamically stable state. The Gibbs free energies of drNGF-TrkA, nnNGF-TrkA, hNGF-TrkA, drNGF, nnNGF, and TrkA were found to be −11.2, −11.9, −11.1, −10.3, −10.8, and −11.6 kJ/mol, respectively, as shown in Figures 9D–F (Sharma and Muniyan, 2024).

## 4 Discussion

In humans, neurogenesis occurs only when the NGF is bound to TrkA. In some cases, there is a lack of NGF in the body owing to either shortage in the synthesis of NGF or reduced processing of proNGF, leading to neurodegenerative conditions like AD and PD. Among the various management options developed for neurodegenerative conditions, exogenous administration of NGF has become the prominent choice for AD (Jönhagen et al., 1998), PD (Olson et al., 1991; 1992; Jönhagen et al., 1998), and traumatic brain injury (Holtzman et al., 1996). The therapeutic potential of NGF has also been tested in neurotrophic keratitis (Bonini et al., 2000), glaucoma (Lambiase et al., 2009a), macular degeneration (Lambiase et al., 2009b), retinitis pigmentosa (Falsini et al., 2016), and pressure ulcers (Bernabei et al., 1999; Landi et al., 2003). However, the potential limitations of using hNGF could be its insufficient oral bioavailability, uncertain pharmacokinetics, suboptimal pharmacological characteristics, limited capacity to penetrate the BBB, shortened half-life, activation of numerous receptors, and multiple effects that still need to be evaluated in the context of other NGF sources like mice and snakes that share structural and functional similarities with hNGF (Skaper, 2008; Lu et al., 2017; Bernardes et al., 2018; Gudasheva et al., 2019; Zhou et al., 2023; Bernardes et al., 2018; Zhou et al., 2023; Lu et al., 2017). Thus, synthetic sNGF forms like drNGF and nnNGF that are structurally and physiochemically similar with identical folding patterns as that of hNGF could be used to induce neuritogenesis.

Various venom-derived drugs have been tested for their pharmacological abilities and are approved by the United States Food and Drug Administration, such as Ancrod as a defibrinogenating agent, Aggrastat as an anticoagulant, L-amino acid oxidase as an antibacterial agent, Nigexine as an antiviral agent, Captopril for hypertension by inhibiting angiotensin-converting enzyme (Vonk et al., 2011), Ximelagatran as a thrombin inhibitor, and Batroxobin to convert fibrinogen to fibrin (El-Aziz et al., 2019). Therefore, it is pertinent to investigate the therapeutic potentials of sNGFs, which appear to exhibit better bioactivity, specificity, and stability than other forms of NGFs. Different sNGFs have already been tested in terms of chondrogenesis, neurite outgrowth, neuroprotection, and tumour growth inhibition through *in vivo* studies (Katzir et al., 2003; Osipov et al., 2014; Lu et al., 2017; Bernardes et al., 2018). A tripeptide consisting of Glu-Val-Trp obtained from *B. atrox* venom has also exhibited neuritogenesis and antiproliferative effects against toxicity produced by MPP+ in PC12 cells and human neuronal cells. Although NGF from *V. russelli russelli* was modelled and docked with TrkA, the dynamics and stability of the complex have not yet been investigated (Islam et al., 2021). In addition, synthetic peptides TNP and HNP were designed following studies based on docking of *V. russelli russelli* NGF with TrkA, and the molecular dynamics simulation of the docked complex exhibited favourable neuritogenesis activity (Islam et al., 2021; Madhubala et al., 2023). Upon NGF binding to TrkA, the NGF-TrkA complex is internalised into the cell cytoplasm, leading to upregulation of the MAPK, Fas, VEGF, and EGF downstream pathways. The neurite-inducing abilities of these proteins and peptides were studied via measurements of the neurite lengths and cellular differentiation.

These complexes also protect against paraquat-induced neurotoxicity in PC12 cells and apoptosis by mitigating the surplus ROS production, oxidative stress, and untimely programmed cell death to aid cell survival. There is overexpression of receptors TrkA and p75 on the surfaces of cancer cell lines like MDA-MD-231 and MCF-7; owing to such overexpression and the fact that NGF is highly specific to its receptors, these complexes can be potentially used as molecular markers. Conversely, non-cancerous cell lines like HEK-293 and L6 show low TrkA and p75 expression levels, highlighting the specificity of NGF toward its receptors (Martins et al., 2015; Bernardes et al., 2018; Islam et al., 2020b; 2020a; 2021; Madhubala et al., 2023). Although an existing study reported the use of a newly designed peptide (EVW) from *B. atrox* as a therapy for neurodegenerative disorders, we focused on the NGF from *D. russelii* as it has greater similarity to hNGF in terms of the protein sequence (Martins et al., 2015). Our work provides more information about hotspot residues that can be explored in the future to develop new variants or mutants capable of supporting enhanced stability; comparatively, the proteins would also have greater stability, high specificity, and wider functional diversity.

NGFs derived from snake species like *B. atrox, V. russelli, N.naja, Agkistrodon piscivorus, Crotalus adamenteus, Naja kaouthia,* and *N. atra* have been explored in many studies but seem to imply similar functions (Levi-Montalcini and Cohen, 1956; Osipov et al., 2013; 2014; Martins et al., 2015; Jin et al., 2017; Lu et al., 2017; Bernardes et al., 2018; Islam et al., 2021). These studies do not report the allergenicity and toxicity of the sNGFs, so subtle differences in the TrkA-binding residues may not produce observable changes in receptor signalling. As NGF binding with TrkA is a prerequisite for inducing neuritogenesis, molecular docking studies were conducted. We would like to acknowledge that the dimerisation of Nerve Growth Factor (NGF) is a well-established paradigm, widely implicated in facilitating stable receptor binding and signalling. However, current literature increasingly suggests that for specific snake venom-derived NGFs, the monomeric form is sufficient to elicit the required biological responses. This functional capacity of the monomer is supported by key findings (Danse and Garnier, 1993): demonstrated that a covalent linkage between NGF monomers in snake venom is highly unlikely, given the high evolutionary conservation of the mature protein. Furthermore (Earl et al., 2006), through mass spectrometry analysis and spot selection in Australian elapid venoms, reported that NGF isoforms approximate the size of a monomeric form, with no evidence for the presence of dimeric species. This study further showed that the partially purified fractions of NGF could induce neurite outgrowth activity, but the presence of a dimeric form could not be detected. This is further corroborated by earlier studies on venom molecular heterogeneity by (Angeletti, 1968), which identified fully active, low-molecular-weight NGF forms that function independently of a dimeric structure. β-NGF monomer from *N. sputatrix,* which is 14 kDa in molecular mass when assayed for neurite activity outgrowth on PC12 cells, exhibited pronounced activity as indicated by (Koh et al., 2004). Collectively, these findings provide a robust biological rationale for our *in silico* study, which focuses on the monomeric NGF-TrkA interaction as the fundamental unit of activity for the selected snake venom mutants. Although some studies have reported the docking of different isoforms of drNGF with TrkA, domain-5 has been explored in our study as the principal domain for binding of sNGF; our docking results show that the binding patterns and sites are similar to those of hNGF. Thus, the possible interactions between sNGFs and TrkA were determined to show conservation of the interacting sites. Then, the binding stabilities were assessed via molecular dynamics simulations since the correlations between the binding affinities and stability characteristics are not always consistent. We found that drNGF exhibited considerable interactions with TrkA compared to nnNGF and hNGF, as indicated by its favourable binding free energy. However, PCA showed the flexibility of these complexes in the order hNGF-TrkA > drNGF-TrkA > nnNGF-TrkA, indicating that nnNGF-TrkA exhibits more compact and restricted motions along its principal eigenvectors.

Although the computational analyses show that drNGF-TrkA and nnNGF-TrkA interactions entail substantial binding free energies, some existing studies have reported sNGF activities with TrkA in the order of recombinant hNGF > nnNGF > drNGF. The enhanced binding free energies observed in the drNGF-TrkA and nnNGF-TrkA interactions may be the key to their stronger binding characteristics. Such binding strength could imply effective receptor activation, stronger stabilisation, and potentially sustained NGF signalling, which require further experimental validation as there are no reports claiming higher TrkA activation for drNGF or nnNGF (Katzir et al., 2003). This flexibility would allow these molecules to fit into the TrkA framework more effectively. The combination of higher binding strength and greater flexibility in drNGF suggests possible evolution towards a specific purpose related to the venom’s effects on receptors rather than the normal signalling functions of neurotrophins. Since sNGFs seemingly bind to TrkA with substantial binding energies, as observed in this *in silico* study, the sNGF molecules may serve as potential structural templates for creating new molecules to bind to TrkA with high affinities while possibly modulating its activities.

The pro-domain present in proNGF displays some regulatory roles in cell proliferation and differentiation, receptor selectivity, and aiding in the folding of mature NGFs (Fahnestock et al., 2004). The processing of proNGF to mature NGF is achieved using furins and proconvertases or through plasmins and matrix metalloproteinases (Fahnestock and Shekari, 2019). Although proNGF does not have a direct role in the NGF-TrkA binding mode, it helps with receptor selectivity; proNGF has a higher affinity to p75 than TrkA in the presence of sortilin, thereby mediating apoptosis. However, higher expressions of TrkA on the cell surfaces or higher levels of mature NGFs can lead to neurogenesis (Lee et al., 2001; Nykjaer et al., 2004; Hempstead, 2006; Armugam et al., 2012).

## 5 Conclusion

The present study was aimed at exhibiting the molecular evidence of interactions between sNGFs and TrkA as being similar to those of hNGF-TrkA. Our results show that drNGF and nnNGF have 87.2% and 79% similarities with hNGF, while the interacting sites with TrkA are also 67.5% and 87.5% similar to those of hNGF, respectively. Following molecular docking of the sNGFs with TrkA, the complexes were subjected to molecular dynamics simulations for a period of 100 ns. These simulations indicate that the sNGFs and hNGF display similar stabilities, with the sNGFs showing stronger interactions. Our computational findings suggest that sNGFs may form stable complexes with TrkA compared to hNGF. However, these predictions require experimental confirmation to establish their validity. The drNGF-TrkA and nnNGF-TrkA complexes exhibited equivalent stabilities to that of hNGF-TrkA, albeit with lower fluctuations. However, the post-simulation mmPBSA analysis shows that both drNGF-TrkA and nnNGF-TrkA exhibit similar stabilities as the hNGF-TrkA complex in terms of their binding energies. The present *in silico* analysis reveals that all three complexes exhibit similar interactions and that there are no major differences for binding with either sNGF or hNGF. Overall, it is observed that there is no change in the amino acid residues interacting with the receptor, either in the monomeric or dimeric form of the growth factor. With the emerging discovery of short peptides which can activate the cell surface growth factor receptors, the study assume more importance, to the tune that it supports the contention that the growth factor can bind to the growth factor receptor in its monomeric state to elicit the required molecular responses/cell signalling events. Our computational assays suggest that sNGFs could be used as functional alternatives to hNGF; however, as this study is purely predictive in nature and entails primary and limited *in vitro* investigations indicating the neurotrophic effects of the sNGFs, detailed experiments are needed to validate their therapeutic potentials. It is also noted that preliminary studies have been conducted in this regard using NGFs from *D. russelii, N. naja kaouthia*, mice, and recombinant human sources on the rat pheochromocytoma PC12 cell line (Katzir et al., 2003).

By characterising the binding mechanisms in sNGFs that demonstrate increased affinities and flexibilities relative to hNGF, this study offers new perspectives on the distinct biological properties of venom-derived NGFs and their potential roles in TrkA regulation. The combined energetic and dynamic analyses suggest that the sNGFs and hNGF may bind to TrkA through different recognition modes, providing a useful basis for further exploration of TrkA modulation. These insights may help inform future efforts on designing receptor-targeted therapeutics. The *in vitro* validations for the binding of sNGFs to TrkA are inferred from the estimates of the outgrowth assays using the PC12 cell line (Katzir et al., 2003). Additionally, cell survival and differentiation assays need to be performed to establish the neuritogenic properties of sNGFs. Similarly, Scatchard analysis, isothermal titration calorimetry, fluorimetry, surface plasmon resonance, and biolayer interferometry are suggested for determining the affinities and binding kinetics of sNGFs to TrkA. These NGF cloning and expression studies are currently being performed in our laboratory to facilitate future functional and structural studies.

## Data Availability

The raw data supporting the conclusions of this article will be made available by the authors without undue reservation.

## Abbreviations

NGF, nerve growth factor; drNGF, *Daboia russelii* nerve growth factor; nnNGF, *Naja naja* nerve growth factor; hNGF, human nerve growth factor; sNGF, snake venom nerve growth factor; TrkA, tropomyosin receptor kinase A; PCA, principal component analysis; mmPBSA, molecular mechanics Poisson–Boltzmann surface area; BBB, blood–brain barrier; FEL, free energy landscape; RMSD, root mean-squared distance; RMSF, root mean-squared fluctuation; AD, Alzheimer’s disease; PD, Parkinson’s disease; ROS, reactive oxygen species; MSA, multiple sequence alignment; Rg, radius of gyration; SASA, solvent accessibility surface area.
