# Correction: Unraveling the molecular basis of snake venom nerve growth factor: human TrkA recognition through molecular dynamics simulation and comparison with human nerve growth factor

**DOI:** 10.3389/fbinf.2026.1897250

**Published:** 2026-07-02

**Authors:** Shrudhi Devi, Gurunathan Jayaraman

**Affiliations:** School of Biosciences and Technology, Vellore Institute of Technology, Vellore, Tamil Nadu, India

**Keywords:** snake venom, nerve growth factor, tyrosine receptor kinase A, neurodegenerative disease, simulation

In the published article, information was incorrectly omitted from the Discussion section. A correction has been made to the section Discussion, Paragraph 4:

“NGFs derived from snake species like *B. atrox, V. russelli, N.naja, Agkistrodon piscivorus, Crotalus adamenteus, Naja kaouthia,* and *N. atra* have been explored in many studies but seem to imply similar functions (Levi-Montalcini and Cohen, 1956; Osipov et al., 2013; 2014; Martins et al., 2015; Jin et al., 2017; Lu et al., 2017; Bernardes et al., 2018; Islam et al., 2021). These studies do not report the allergenicity and toxicity of the sNGFs, so subtle differences in the TrkA-binding residues may not produce observable changes in receptor signalling. As NGF binding with TrkA is a prerequisite for inducing neuritogenesis, molecular docking studies were conducted. We would like to acknowledge that the dimerisation of Nerve Growth Factor (NGF) is a well-established paradigm, widely implicated in facilitating stable receptor binding and signalling. However, current literature increasingly suggests that for specific snake venom-derived NGFs, the monomeric form is sufficient to elicit the required biological responses. This functional capacity of the monomer is supported by key findings (Danse and Garnier, 1993): demonstrated that a covalent linkage between NGF monomers in snake venom is highly unlikely, given the high evolutionary conservation of the mature protein. Furthermore (Earl et al., 2006), through mass spectrometry analysis and spot selection in Australian elapid venoms, reported that NGF isoforms approximate the size of a monomeric form, with no evidence for the presence of dimeric species. This study further showed that the partially purified fractions of NGF could induce neurite outgrowth activity, but the presence of a dimeric form could not be detected. This is further corroborated by earlier studies on venom molecular heterogeneity by (Angeletti, 1968), which identified fully active, low-molecular-weight NGF forms that function independently of a dimeric structure. β-NGF monomer from *N. sputatrix,* which is 14 kDa in molecular mass when assayed for neurite activity outgrowth on PC12 cells, exhibited pronounced activity as indicated by (Koh et al., 2004). Collectively, these findings provide a robust biological rationale for our *in silico* study, which focuses on the monomeric NGF-TrkA interaction as the fundamental unit of activity for the selected snake venom mutants. Although some studies have reported the docking of different isoforms of drNGF with TrkA, domain-5 has been explored in our study as the principal domain for binding of sNGF; our docking results show that the binding patterns and sites are similar to those of hNGF. Thus, the possible interactions between sNGFs and TrkA were determined to show conservation of the interacting sites. Then, the binding stabilities were assessed via molecular dynamics simulations since the correlations between the binding affinities and stability characteristics are not always consistent. We found that drNGF exhibited considerable interactions with TrkA compared to nnNGF and hNGF, as indicated by its favourable binding free energy. However, PCA showed the flexibility of these complexes in the order hNGF-TrkA > drNGF-TrkA > nnNGF-TrkA, indicating that nnNGF-TrkA exhibits more compact and restricted motions along its principal eigenvectors.”

A correction has been made to the section Conclusion, Paragraph 1:

“The present *in silico* analysis reveals that all three complexes exhibit similar interactions and that there are no major differences for binding with either sNGF or hNGF. Overall, it is observed that there is no change in the amino acid residues interacting with the receptor, either in the monomeric or dimeric form of the growth factor. With the emerging discovery of short peptides which can activate the cell surface growth factor receptors, the study assume more importance, to the tune that it supports the contention that the growth factor can bind to the growth factor receptor in its monomeric state to elicit the required molecular responses/cell signalling events.Our computational assays suggest that sNGFs could be used as functional alternatives to hNGF; however, as this study is purely predictive in nature and entails primary and limited *in vitro* investigations indicating the neurotrophic effects of the sNGFs, detailed experiments are needed to validate their therapeutic potentials. It is also noted that preliminary studies have been conducted in this regard using NGFs from *D. russelii, N. naja kaouthia,* mice, and recombinant human sources on the rat pheochromocytoma PC12 cell line (Katzir et al., 2003).”

The original article has been updated.

