# Supplementary material for: Unraveling the molecular basis of snake venom nerve growth factor: human TrkA recognition through molecular dynamics simulation and comparison with human nerve growth factor
Source: Front Bioinform. 2025 Oct 24;5:1674791. doi: 10.3389/fbinf.2025.1674791 (PMC12592128; doi:10.3389/fbinf.2025.1674791)
Supplement: Supplementary file 1 [file Supplementaryfile1.docx]

**Unravelling the Molecular Basis for snake venom NGF - human TrkA recognition through Molecular Dynamics Simulation and comparison with Human NGF**

Shrudhi Devi, Gurunathan Jayaraman*

School of Biosciences and Technology, Vellore Institute of Technology, Vellore 632014, Tamil Nadu, India.

*Corresponding author:

**G. Jayaraman**

School of Biosciences and Technology

Vellore Institute of Technology

Vellore 632014, India

Email: [gjayaraman@vit.ac.in](mailto:gjayaraman@vit.ac.in)

Orchid id: <https://orcid.org/0000-0001-5729-2719>

**Supplementary information for NGF-NGFR analysis:**

## Similarity and Identity index :

**TABLE S1** Similarity and Identity index of all species of snake venom NGF with respect to hNGF

| **Si. No** | **Species name** | **Uniprot Accession number** | **Identity %** | **Similarity %** |
| --- | --- | --- | --- | --- |
| 1 | *Agkistrodon contortrix contortrix* | B8QCG6 | 71.4 | 87.4 |
| 2 | *Azemiops feae* | Q2XXL6 | 70.1 | 87.2 |
| 3 | *Acrochordus granulatus* | B8QCG4 | 49.6 | 59 |
| 4 | *Agkistrodon piscivorus* | A0A194ARS6 | 72.6 | 88.9 |
| 5 | *Anilius scytale* | B8QCG8 | 49.6 | 59.8 |
| 6 | *Bitis gabonica* | Q6T6T1 | 22.7 | 27.7 |
| 7 | *Boaedon fuliginosus* | B8QCJ0 | 48.7 | 59.8 |
| 8 | *Bothrops cotiara* | P0DMG7 | 11.8 | 15.1 |
| 9 | *Bothrops jararacussu* | Q90W38 | 73.5 | 88.9 |
| 10 | *Brachyurophis roperi* | R4FI83 | 65.8 | 78.6 |
| 11 | *Bungarus fasciatus* | V9I1A1 | 69.2 | 82.9 |
| 12 | *Bungarus multicinctus* | P34128 | 72.6 | 85.5 |
| 13 | [*Cacophis squamulosus*](https://www.uniprot.org/taxonomy/505434) | R4G7F6 | 19.9 | 23.8 |
| 14 | *Calliophis bivirgatus* | A0A898IKL9 | 65 | 79.5 |
| 15 | *Crotalus adamanteus* | J3SDV8 | 72.6 | 88 |
| 16 | *Crotalus durissus terrificus* | Q9DEZ9 | 72.6 | 88 |
| 17 | [*Crotalus horridus*](https://www.uniprot.org/taxonomy/35024) | T1E3D1 | 72.6 | 88 |
| 18 | [*Cryptophis nigrescens*](https://www.uniprot.org/taxonomy/292442) | Q1W7Q6 | 70.1 | 82.9 |
| 19 | [*Cylindrophis ruffus*](https://www.uniprot.org/taxonomy/186578) | B8QCH9 | 49.6 | 59.8 |
| 20 | *Daboia russelii* | P30894 | 70.9 | 87.2 |
| 21 | *Demansia vestigiata* | A6MFL5 | 73.5 | 85.5 |
| 22 | *Denisonia devisi* | R4FJL1 | 63.9 | 76.5 |
| 23 | *Diadophis punctatus* | B8QCI1 | 48.7 | 59.8 |
| 24 | *Drysdalia coronoides* | F8RKW5 | 71.8 | 82.1 |
| 25 | [*Echiopsis curta*](https://www.uniprot.org/taxonomy/529692) | R4FID6 | 70.9 | 84.6 |
| 26 | *Echis coloratus* | A0A081DUA8 | 72.6 | 88 |
| 27 | *Echis ocellatus* | P0DMD1 | 11.5 | 14.3 |
| 28 | [*Furina ornata*](https://www.uniprot.org/taxonomy/529697) | R4G2V1 | 70.1 | 80.3 |
| 29 | *Heterodon platirhinos* | B8QCI5 | 47.9 | 58.1 |
| 30 | [*Hoplocephalus bungaroides*](https://www.uniprot.org/taxonomy/83403) | R4G2I4 | 70.9 | 83.8 |
| 31 | *Hoplocephalus stephensii* | Q3HXY0 | 70.9 | 83.8 |
| 32 | *Imantodes cenchoa* | B8QCI7 | 30.5 | 36.9 |
| 33 | *Laticauda colubrina* | B8QCJ1 | 49.6 | 59.8 |
| 34 | *Liotyphlops albirostris* | B8QCJ4 | 45.3 | 55.6 |
| 35 | *Lycophidion capense* | B8QCJ6 | 30.5 | 38 |
| 36 | *Macrovipera lebetina* | P25428 | 58.1 | 71.5 |
| 37 | *Micrurus fulvius* | U3EPH5 | 70.1 | 82.1 |
| 38 | *Micrurus tener* | A0A194AT78 | 70.1 | 82.1 |
| 39 | *Naja atra* | P61898 | 65.5 | 79 |
| 40 | *Naja kaouthia* | P61899 | 65.5 | 79 |
| 41 | *Naja naja* | P01140 | 65.8 | 78.3 |
| 42 | *Natrix natrix* | B8QCJ9 | 47 | 57.3 |
| 43 | *Notechis scutatus scutatus* | Q3HXY7 | 68.1 | 79.8 |
| 44 | *Naja sputatrix* | Q5YF90 | 76.1 | 88.9 |
| 45 | [*Opheodrys aestivus*](https://www.uniprot.org/taxonomy/186591) | A0A081DUB8 | 13.9 | 16.6 |
| 46 | [*Oxyuranus microlepidotus*](https://www.uniprot.org/taxonomy/111177) | Q3HXZ1 | 73.5 | 84.6 |
| 47 | [*Ovophis okinavensis*](https://www.uniprot.org/taxonomy/8769) | T2HPR2 | 73.5 | 88 |
| 48 | *Oxyuranus scutellatus scutellatus* | Q3I5F4 | 73.5 | 84.6 |
| 49 | [*Pantherophis guttatus*](https://www.uniprot.org/taxonomy/94885) | A0A081DUC2 | 62.4 | 75.2 |
| 50 | *Parasuta nigriceps* | H8PG96 | 83.8 | 83.8 |
| 51 | [*Protobothrops elegans*](https://www.uniprot.org/taxonomy/88086) | A0A077L7X3 | 74.4 | 88.9 |
| 52 | [*Protobothrops flavoviridis*](https://www.uniprot.org/taxonomy/88087) | B1Q3K2 | 74.4 | 88.9 |
| 53 | [*Pseudechis australis*](https://www.uniprot.org/taxonomy/8670) | Q3HXY3 | 70.9 | 81.2 |
| 54 | [*Pseudechis papuanus*](https://www.uniprot.org/taxonomy/61265) | A0A1D8BAW9 | 79.2 | 80.3 |
| 55 | *Pseudechis porphyriacus* | Q3HXY4 | 56.2 | 66.5 |
| 56 | [*Pseudonaja modesta*](https://www.uniprot.org/taxonomy/340912) | R4FIR0 | 65.4 | 78 |
| 57 | [*Pseudonaja textilis*](https://www.uniprot.org/taxonomy/8673) | Q3HXY9 | 74.4 | 87.2 |
| 58 | [*Python regius*](https://www.uniprot.org/taxonomy/51751) | A0A081DUC4 | 76.9 | 89.7 |
| 59 | [*Rena humilis*](https://www.uniprot.org/taxonomy/711330) | B8QCJ2 | 31 | 37.4 |
| 60 | [*Sistrurus miliarius barbouri*](https://www.uniprot.org/taxonomy/8759) | A0A194AQ56 | 71.8 | 87.2 |
| 61 | *Sistrurus tergeminus* | A0A194ARN2 | 72.6 | 87.2 |
| 62 | [*Sonora semiannulata*](https://www.uniprot.org/taxonomy/186602) | B8QCK3 | 21.4 | 27.3 |
| 63 | [*Trimorphodon biscutatus*](https://www.uniprot.org/taxonomy/338818) | B8QCK6 | 30.5 | 36.9 |
| 64 | [*Tropidechis carinatus*](https://www.uniprot.org/taxonomy/100989) | Q3HXX4 | 71.8 | 84.6 |
| 65 | [*Vipera anatolica senliki*](https://www.uniprot.org/taxonomy/2604287) | A0A6G5ZVU9 | 73.5 | 88 |
| 66 | [*Vipera ursinii*](https://www.uniprot.org/taxonomy/103942) | V9I168 | 73.5 | 88 |
| 67 | [*Walterinnesia aegyptia*](https://www.uniprot.org/taxonomy/64182) | V9I1F9 | 69.2 | 82.1 |
| 68 | [*Xenopeltis unicolor*](https://www.uniprot.org/taxonomy/196253) | B8QCL2 | 49.6 | 59.8 |

## **Physiochemical characteristics:**

The physiochemical properties of drNGF and nnNGF as predicted by various tools is given below in Table S2. There was neither the presence of any repeats nor transmembrane domain as predicted by the Radar and Phobius tool respectively. The protein stability was predicted to be more than 10 hours in *E.coli* by the tool Protparam while Pfam tool predicted that sNGF is able to perform the same neuritogenesis property as that of hNGF.

**TABLE S2** Physiochemical properties of drNGF and nnNGF.

| **Property** | **drNGF** | **nnNGF** | **Tool used** |
| --- | --- | --- | --- |
| Amino acid residues | 117 | 116 | ProtParam |
| Molecular size | 13283 | 13063 | ProtParam |
| Net charge | Positive | Negative | ProtParam |
| Melting point | >65 °C | 55-65 °C | Tm predictor |
| Solubility | 0.472 | 0.567 | Protein-Sol |
| Half-life | >10 hours in *E.coli* | >10 hours in *E.coli* | ProtParam |
| pI | 8.84 | 6.08 | ProtParam |
| Instability index | 22.14 | 26.52 | ProtParam |
| Hydrophobicity | -0.49 | -0.584 | ProtParam |
| Aliphatic index | 69.01 | 59.57 | ProtParam |

### **Solvent Accessibility Properties by SPIDDER:**

Prediction of the interaction sites of *drNGF* and *nnNGF:*

### ***Daboia russellii* (drNGF):** The amino acid residues that are accessible to the solvent or binding are H1, P2, V3, H4, N5, Q6, G7, E8, F9, S10, V11, W18, N21, M28, V32, V45, Y46, K47, Q48, Y49, F50, F51, G67, I68, W73, V84, R85, L87, S95, W96, F98, R100, T103, I109, R111, N113, F116 and G117.

### ***Naja naja* (nnNGF)*:*** The amino acid residues that are accessible to the solvent or binding are P4, V5, H6, N7, L8, G9, E10, H11, W20, I29, D43, N44, V46, Y47, E49, Y50, G68, I69, I85, W97, F99, R101, I110 and T111.

## **RMSF values of the active site residues of the complexes:**

**TABLE S3:** The RMSF values of the active site residues of hNGF-trkA, drNGF-trkA, nnNGF-trkA, drNGF and nnNGF are given.

| **Active site residues** | **hNGF-trkA (Bound form)** | **drNGF-trkA (Bound form)** | **nnNGF-trkA (Bound form)** | **drNGF (Apo form)** | **nnNGF (Apo form)** |
| --- | --- | --- | --- | --- | --- |
| H4 | 0.60 | 1.01 | 0.36 | 1.18 | 0.62 |
| R9 | 0.67 | 0.37 | 0.37 | 0.52 | 0.45 |
| E11 | 0.54 | 0.16 | 0.21 | 0.24 | 0.31 |
| W21 | 0.21 | 0.13 | 0.15 | 0.17 | 0.15 |
| R59 | 0.18 | 0.13 | 0.13 | 0.15 | 0.13 |
| H84 | 0.12 | 0.11 | 0.11 | 0.12 | 0.12 |
| R103 | 0.13 | 0.10 | 0.12 | 0.11 | 0.09 |

**TABLE S4** Average values of Molecular Dynamics Simulation Results. The RMSD and RMSF values of sNGF is lesser than that of hNGF indicating the stable nature of sNGF over hNGF.

| **Complex** | **Name** | **RMSD (nm)** | **RMSD of the side chain (nm)** | **RMSF (nm)** | **Rg (nm)** | **SASA (nm^2^)** | **Number of H-bond** |
| --- | --- | --- | --- | --- | --- | --- | --- |
| 1 | drNGF-TrkA receptor | 0.59 | 0.70 | 0.21 | 2.22 | 130.43 | 126.76 |
| 2 | nnNGF-TrkA receptor | 0.61 | 0.67 | 0.22 | 2.23 | 130.07 | 118.94 |
| 3 | hNGF-TrkA receptor | 0.59 | 0.69 | 0.27 | 2.11 | 117.20 | 126.13 |
| 4 | drNGF | 0.34 | 0.46 | 0.24 | 2.02 | 67.01 | 68.47 |
| 5 | nnNGF | 0.42 | 0.55 | 0.26 | 1.99 | 65.96 | 66.93 |
| 6 | TrkA receptor | 0.81 | 0.82 | 0.33 | 1.63 | 78.16 | 51.17 |

**Figures:**


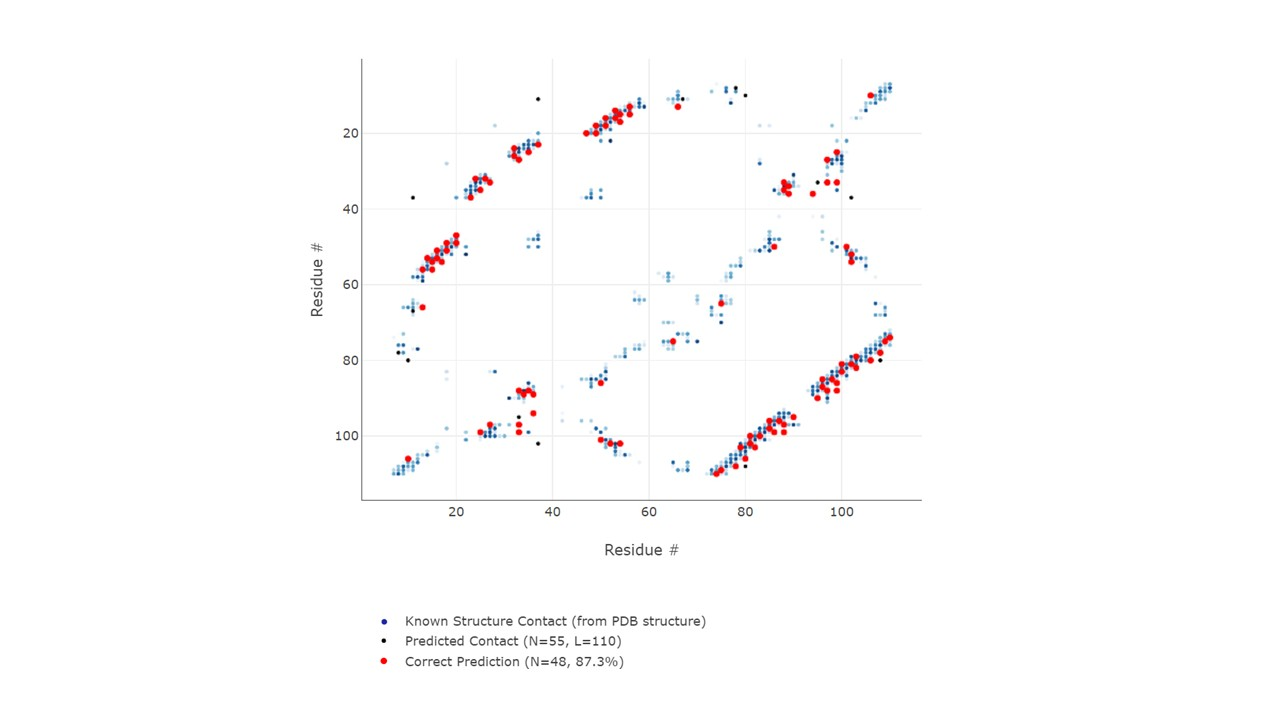


**FIGURE S1** Contact map of the evolutionary coupled residues. Red coloured dots represent the contact residues that are experimentally determined while the blue coloured ones are the predicted ones

**
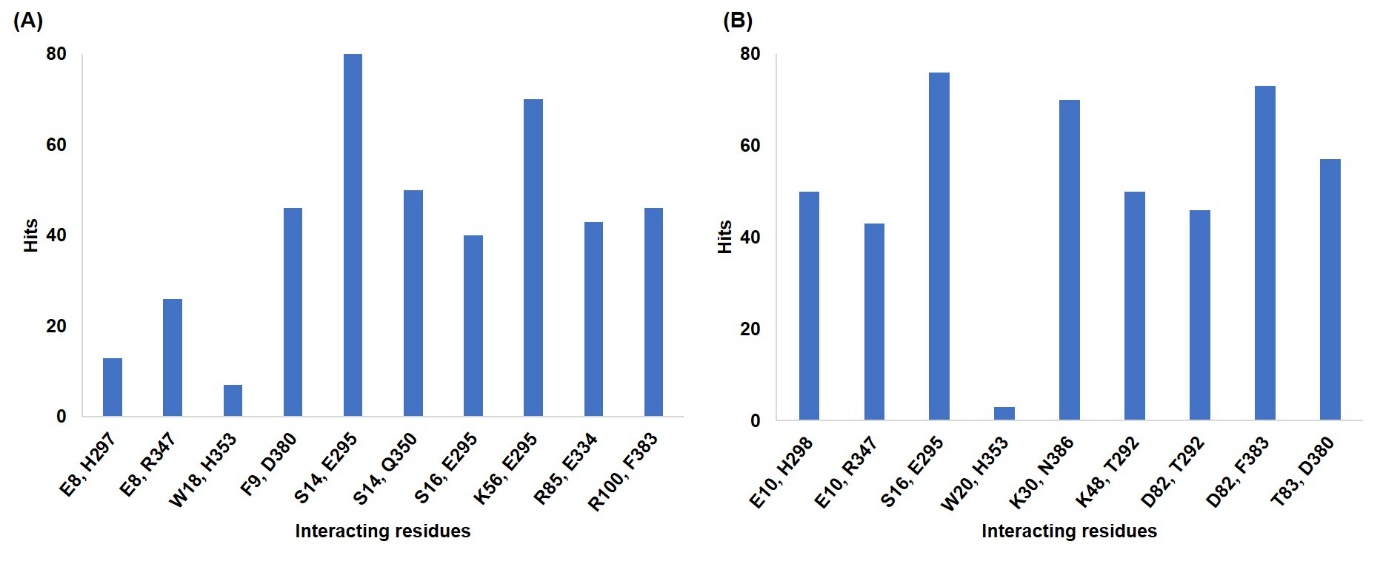
**

**FIGURE S2(A)** Statistical analysis of the docked complexes of drNGF-TrkA and (B) for nnNGF-TrkA. In (A), in the interaction between drNGF-TrkA, there are a higher number of complexes exhibiting bonding between (S14, E295). In the same way nnNGF-TrkA also exhibits same type of interaction (S16, E295) multiple times as shown in FIGURE S2(B)


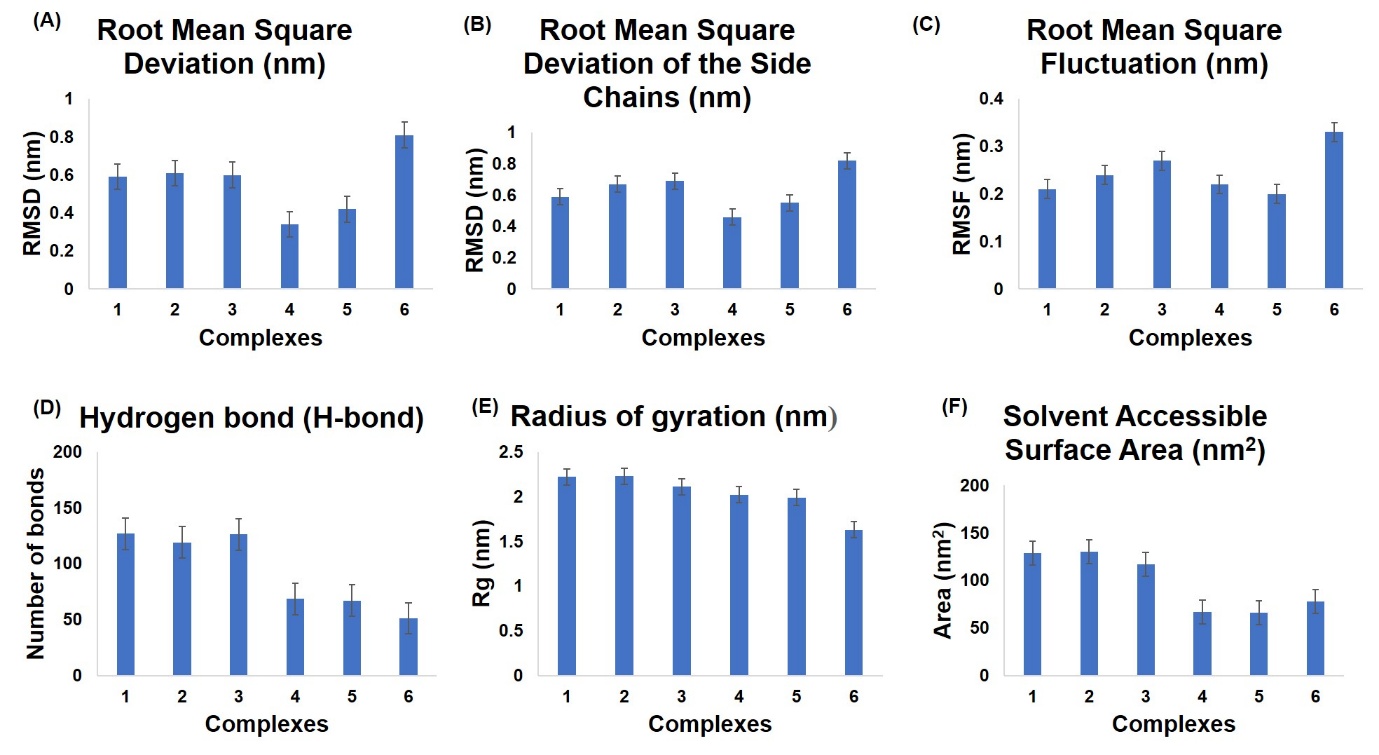


**FIGURE S3** Statistical analysis of the average values of (A): RMSD of backbone, (B): RMSD of the side chain, (C): RMSF values between NGF-TrkA complex, (D): Radius of gyration, (E): SASA and (F):number of hydrogen bond between NGF-TrkA complex. Complex 1: drNGF-TrkA, Complex 2: nnNGF-TrkA, Complex 3: hNGF-TrkA complex, Complex 4: drNGF Complex 5: nnNGF and Complex 6: TrkA receptor
